# A Case Report of Sjögren’s Syndrome and Rheumatoid Arthritis: The Role of Physiotherapy in Enhancing Quality of Life

**DOI:** 10.7759/cureus.55411

**Published:** 2024-03-02

**Authors:** Sawari S Bhagwatkar, Pallavi Harjpal, Prajyot Ankar

**Affiliations:** 1 Physiotherapy, Ravi Nair Physiotherapy College, Datta Meghe Institute of Higher Education and Research, Wardha, IND; 2 Neurophysiotherapy, Ravi Nair Physiotherapy College, Datta Meghe Institute of Higher Education and Research, Wardha, IND

**Keywords:** physiotherapy rehabilitation, quality of life, sjögren's syndrome, sacroiliac joint dysfunction, rheumatoid arthritis

## Abstract

Rheumatoid arthritis (RA) is a systemic autoimmune disease with profound effects on joints and extra-articular organs. This case report explores the complex treatment approach for a 54-year-old female patient who is dealing with the dual diagnosis of RA and Sjogren's syndrome (SS). RA primarily involves joint inflammation and morning stiffness leading to significant disability, while SS, another autoimmune condition, manifests with autoantibodies and lymphocytic infiltration affecting exocrine glands. The patient presented with joint and low back pain, alongside reduced mobility, portraying a complex clinical picture. Physiotherapy played a crucial role in addressing the diverse symptoms exhibited by the patient. Treatment involved Mulligan mobilization targeting sacroiliac joint dysfunction, laser therapy for pain relief, and tailored exercises focusing on joint mobility and muscle strength. Progress was monitored using the Rheumatoid Arthritis Disease Activity Index (RADAI-5) and overall quality of life assessments. Significant improvements were observed post-rehabilitation including reduced pain levels, increased joint range of motion, increased muscle strength, and enhanced sacroiliac mobility. These positive outcomes highlight the efficacy of physiotherapy in managing autoimmune rheumatic disorders. Collaboration between healthcare professionals particularly rheumatologists and physiotherapists is essential for comprehensive patient care. This case emphasizes the importance of adopting a holistic approach to managing autoimmune disorders. Physiotherapy emerges as a pivotal component in alleviating symptoms and enhancing physical function underscoring its integration into the multidisciplinary care framework for individuals facing the challenges of autoimmune rheumatic disorders.

## Introduction

Rheumatoid arthritis (RA) is a systemic autoimmune illness linked to a persistent inflammatory process [1]. Both joints and extra-articular organs such as the kidney, heart, digestive tract, lung, skin, eyes, and nervous system can be harmed by RA [2]. In the industrialized world, 0.5-1% of people suffer from RA, a chronic inflammatory and degenerative joint disease that frequently results in severe disability and a decline in their quality of life [3]. Sjögren's syndrome (SS), adult-onset scleroderma, polymyositis, psoriatic arthritis, spondylarthritis, scleroderma, and other conditions are included in the diverse group of autoimmune rheumatic illnesses [4]. Articular and periarticular symptoms include joint swelling and tenderness while palpating, stiffness experienced in the morning, and significant restriction of movement in the affected joints. Despite variations in the clinical manifestation of RA, the prevailing observation is the gradual onset of pain accompanied by symmetrical inflammation in small joints. An early indication of RA is typically stiffness in and around the joints that lasts for at least one hour before improving to its maximum. The lumbar spine, sacroiliac, and distal interphalangeal joints are unlikely to be impacted yet any joint including the cricoarytenoid joint may be [5].

The production of many autoantibodies in the bloodstream and lymphocytic infiltration of exocrine glands and other organs are symptoms of SS, a chronic autoimmune rheumatic condition [6]. Common symptoms encompass fatigue, pain in the muscles and bones, parched mouth and eyes, and enlargement of the primary salivary glands. The sicca syndrome, named after the Latin word siccus, which means dry or thirsty is a condition in which there is generally whole-body dryness due to the gradual and cumulative destruction and failure of exocrine glands [7]. The majority of people with SS, an autoimmune disease that progresses slowly, are middle-aged women, and the female-to-male ratio can reach 9:1. When a main disease, usually another autoimmune connective tissue disease, manifests first, a version of the syndrome known as secondary Sjogren's syndrome happens. Scleroderma, polymyositis, RA, lymphoma, and systemic lupus erythematosus are among the primary diseases that are commonly associated with SS [8]. Moreover, chronic autoimmune conditions like RA and SS can often lead to secondary musculoskeletal issues such as low back pain (LBP) due to altered biomechanics, inflammation, and reduced mobility. LBP can be reduced by proper physiotherapy intervention.

The sacroiliac joint (SIJ) is the largest axial joint found in the human body. These are diarthrodial synovial joints, which connect the iliac bones to the sacrum, have an auricular form, and are found in the pelvis [9]. One major health issue that significantly affects both healthcare expenses as well as quality of life is LBP [10]. A common cause of LBP is sacroiliac joint dysfunction (SIJD). In 16-30% of LBP patients, SIJD is present. The cause of pain originating from the SIJ remains unclear. Myofascia, enthesopathy, fractures, and ligamentous injuries are examples of extra-articular sources of dysfunction. Obstetrics, scoliosis, trauma, leg length disparity, aberrant gait patterns, and lumbar fusion surgery with sacrum fixation are among the risk factors [11]. SIJD can also have an idiopathic onset or be the consequence of direct trauma. Medical and physical therapy methods can be used to treat SIJD. The most successful method and one that is most frequently employed in physical therapy clinics, manipulation is one of the effective physiotherapy strategies for lowering pain and impairment related to SIJD [12]. Pain can be effectively reduced, and functionality can be preserved with physical treatment, which also involves dynamic exercise therapy, hand exercises, hydrotherapy, patient education, joint protection training using behavioral approaches, and cognitive-behavioral treatment (for those with lower psychological status) [13]. Exercise treatment may be suggested as a good means of improving physical capability and lowering disease activity [14]. Patients with secondary SS experience fatigue which can be managed with physical therapy. The first line of treatment for fatigued patients is a low-impact aerobic exercise regimen.

## Case presentation

Patient information

A 54-year-old female patient presented herself at Acharya Vinoba Bhave Rural Hospital (AVBRH) seeking medical attention for pain experienced in her lower back and joints (hips, knees, ankles, shoulder, elbow, metacarpophalangeal, proximal interphalangeal, and wrist joints bilateral). The patient has a confirmed diagnosis of RA for the past 12 years and a surgical history of total abdominal hysterectomy (THA). Following the THA, the patient experienced recurrent fever and infections leading her to seek treatment at a private hospital in Delhi. Diagnostic tests, including ANA immunofluorescence and ANA-LIA serum, revealed that the patient also has SS. As a result, the healthcare provider recommended the administration of non-steroidal anti-inflammatory drugs to the patient. Presently, the patient is experiencing joint pain and LBP, leading to her admission to AVBRH on December 24, 2023. The pain began gradually and worsened with prolonged standing, walking, knee bending, and writing, but improved with rest and medication. Investigations, including X-rays and an MRI, identified degenerative changes in the L4-L5 region. As a result, the patient has been directed to the physiotherapy department for further treatment.

Clinical findings

The patient provided verbal consent prior to the examination, demonstrating that the patient was conscious and had full awareness of their surroundings including person, place, and time. On observation, the patient presented with reduced mobility, flexibility, and strength. The patient also presented a waddling gait pattern. On examination, there was reduced range of motion of the hip, knee, ankle, metacarpophalangeal, proximal interphalangeal, and wrist joint and reduced muscle strength of same. On assessment, the Gaenslen test was performed and the test was positive for SIJD. Table 1 demonstrates the range of motion for the lower limb and upper limb and Table 2 demonstrates manual muscle testing for the lower limb and upper limb.

**Table 1:** Range of motion (ROM) for the bilateral lower limb and bilateral upper limb

Joint	Right (Affected)	Left (Affected)
Shoulder		
Flexion	0^0^-120^0^	0^0^-90^0^
Extension	0^0^-60^0^	0^0^-50^0^
Abduction	0^0^-124^0^	0^0^-110^0^
Medial rotation	0^0^-70^0^	0^0^-55^0^
Lateral rotation	0^0^-80^0^	0^0^-70^0^
Elbow		
Flexion	0^0^-120^0^	0^0^-120^0^
Extension	120-0^0^	120-0^0^
Wrist		
Flexion	0^0^-70^0^	0^0^-65^0^
Extension	0^0^-55^0^	0^0^-45^0^
Radial deviation	0^0^-15^0^	0^0^-15^0^
Ulnar deviation	0^0^-20^0^	0^0^-25^0^
Hip		
Flexion	0^0^-80^0^	0^0^-70^0^
Extension	0^0^-24^0^	0^0^-20^0^
Abduction	0^0^-40^0^	0^0^-35^0^
Adduction	0^0^-20^0^	0^0^-20^0^
External rotation	0^0^-35^0^	0^0^-20^0^
Internal rotation	0^0^-35^0^	0^0^-25^0^
Knee		
Flexion	0^0^-90^0^	0^0^-80^0^
Extension	90-0^0^	80-0^0^
Ankle		
Dorsiflexion	0^0^-10^0^	0^0^-15^0^
Plantarflexion	0^0^-25^0^	0^0^-30^0^
Inversion	0^0^-20^0^	0^0^-25^0^
Eversion	0^0^-10^0^	0^0^-10^0^

**Table 2:** Manual muscle testing (MMT) for the bilateral lower limb and bilateral upper limb This is using manual muscle testing as per Medical Research Council grading [15].

Muscles	Right (Affected)	Left (Affected)
Shoulder		
Flexors	3 out of 5	3 out of 5
Extensors	3 out of 5	3 out of 5
Abductors	3 out of 5	3 out of 5
Medial rotators	3 out of 5	3 out of 5
Lateral rotators	3 out of 5	3 out of 5
Elbow		
Flexors	3 out of 5	3 out of 5
Extensors	3 out of 5	3 out of 5
Wrist		
Flexors	3 out of 5	3 out of 5
Extensors	2 out of 5	2 out of 5
Radial deviators	3 out of 5	3 out of 5
Ulnar deviators	3 out of 5	3 out of 5
Hip		
Flexors	3 out of 5	3 out of 5
Extensors	3 out of 5	3 out of 5
Abductors	3 out of 5	3 out of 5
Adductors	3 out of 5	3 out of 5
Internal rotators	3 out of 5	3 out of 5
External rotators	3 out of 5	3 out of 5
Knee		
Flexors	3 out of 5	3 out of 5
Extensors	3 out of 5	3 out of 5
Ankle		
Dorsiflexors	3 out of 5	3 out of 5
Plantar flexors	3 out of 5	3 out of 5
Invertors	2 out of 5	3 out of 5
Evertors	2 out of 5	2 out of 5

Clinical diagnosis

**Figure 1:**
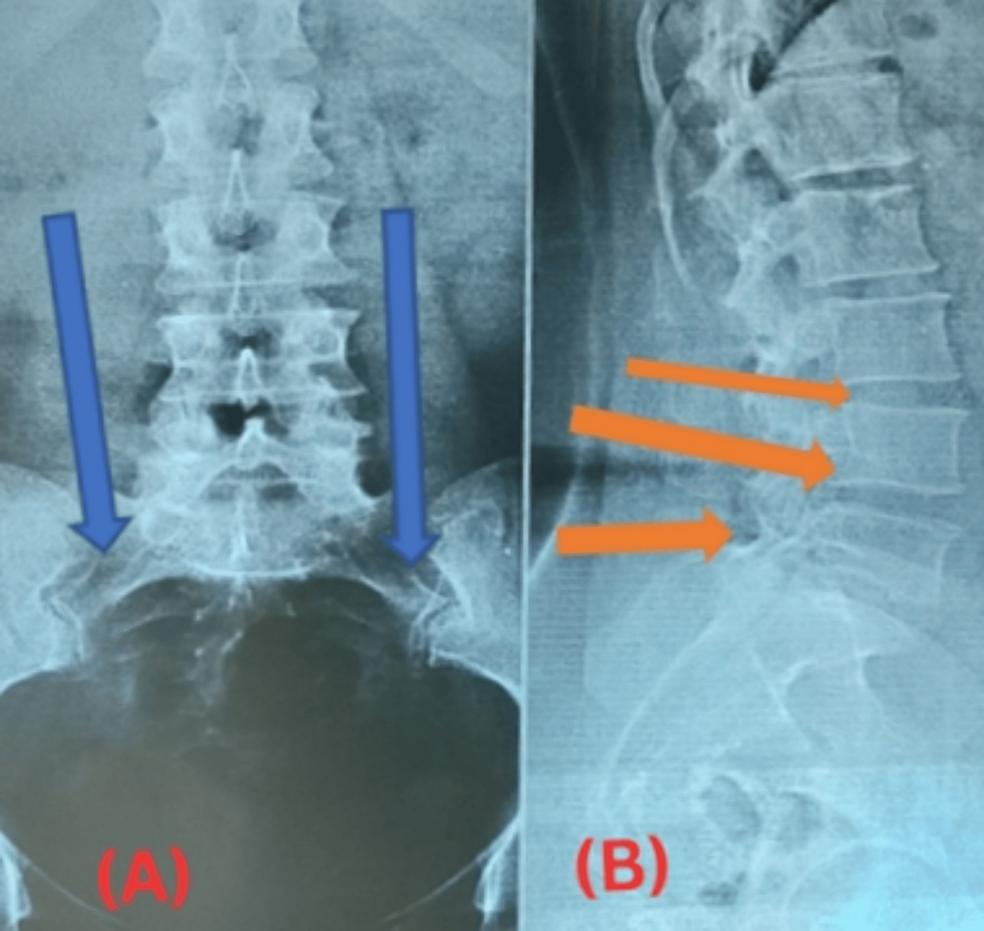
(A) Blue arrows demonstrate SI joint degeneration. (B) Orange arrows demonstrate mild osteophytic change with end plate degeneration SI: Sacroiliac

Diagnostic procedures like histo biopsy salivary gland were done which revealed SS, ANA (Anti-Nuclear Antibody) immunofluorescence which was positive with Hep-2. The ANA-LIA serum test was done which was positive (++) for SS-A/Ro60, SS-ARo52, and SS-B/La and the Anti-CCP antibody test was also positive. X-rays of the spine and sacroiliac joint were done, findings of which are described in Figure 1.

Physiotherapy interventions

**Figure 2:**
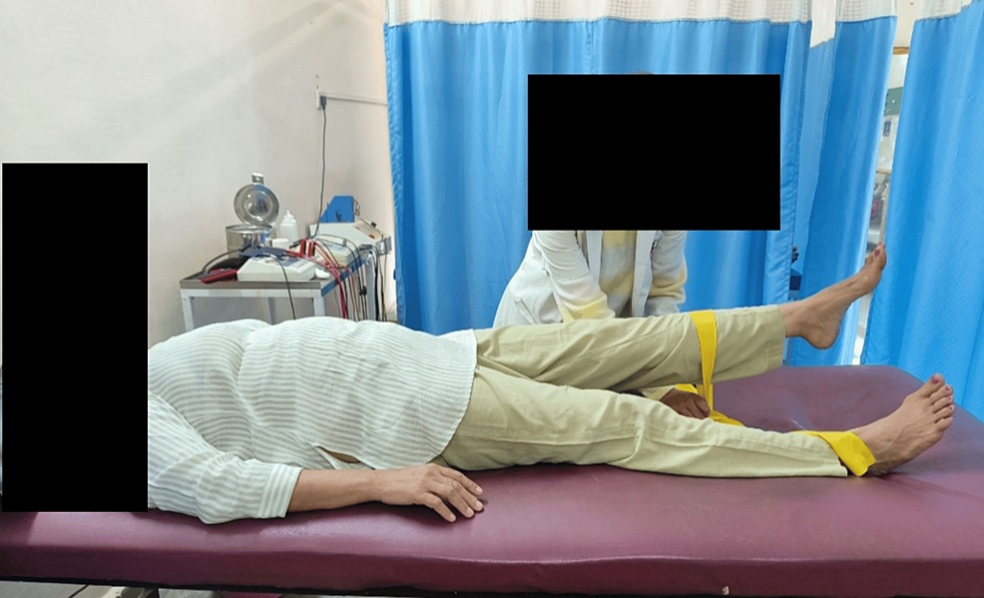
Straight leg raise with yellow theraband (providing less resistance)

**Figure 3:**
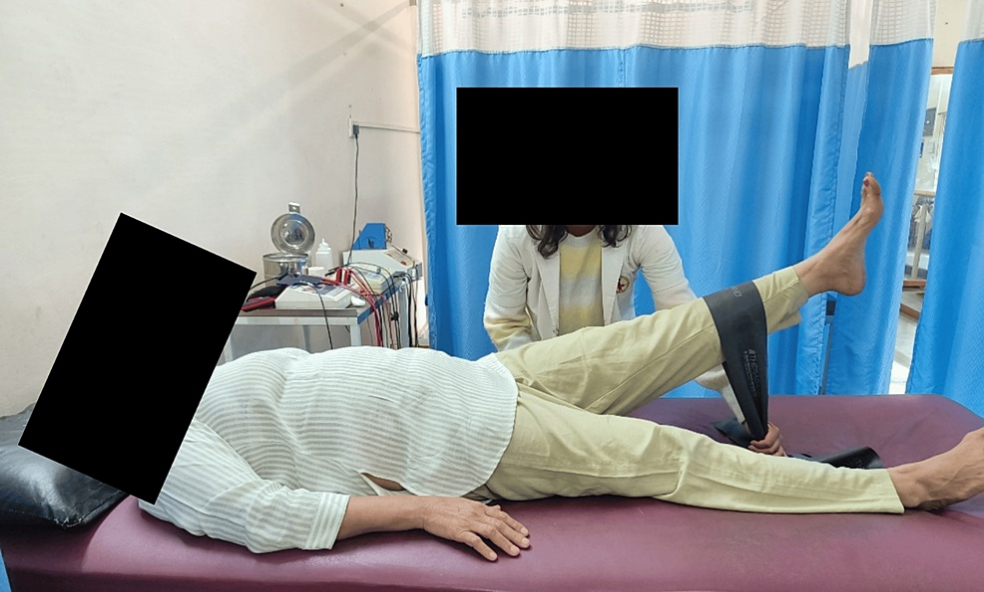
Progression of straight leg raise with black theraband (providing higher resistance)

Table 3 demonstrates physiotherapy interventions, Figure 2 demonstrates straight leg raise with yellow theraband which provides less resistance, and Figure 3 demonstrates progression of straight leg raise with black theraband which provides higher resistance.

**Table 3:** Physiotherapy intervention [14]

Problems identified	Physiotherapeutic goals	Intervention
Lack of awareness regarding physiotherapy treatment	Patient and caregiver education.	Educating patients about recuperation processes, physiological and psychological agony processes, methods of relaxation, and cognitive strategies for coping and managing pain.
The anterior pelvic tilting angle has been observed to be elevated, accompanied by the presence of pain and a decrease in sacroiliac mobility.	To reduce the angle of anterior pelvic tilting, alleviate pain, and enhance sacroiliac mobility	Mulligan mobilization with movement: 1.The therapist used the posterior innominate approach on a patient who was lying prone. He put his hand's thenar eminence on the opposite ilium's slightly projecting posterior border and pushed it away from him. The individual was instructed to perform extension while lying down for up to three sets of ten repetitions, as long as there was no pain. 2. When the therapist employed the anterior innominate technique, the patient was positioned in prone lying. He used the edge of one hand to secure the sacrum, and he placed the fingers of his opposite hand beneath the opposing ASIS. The patient performed ten extensions per set and up to three sets, as long as they were pain-free, while the therapist pulled the ilium up on the sacrum and held this posture.
Pain in low back and proximal joints	To reduce pain	LASER therapy
Tightness of sacroiliac joint musculature	To reduce tightness of sacroiliac joint musculature and also SI joint pain	Sacroiliac Joint Stretches: 1. With the hip flexed to 70 to 80 degrees and the knee bent to around 90 degrees, the patient was positioned in side-lying. After that, the patient's trunk was turned as much as was comfortable towards the top side. The patient was instructed to lift their leg into internal rotation and hip abduction while restraining themselves for five seconds from the researcher or their companion. The trainer gently applied excessive pressure to the patient's trunk rotation while instructing them to breathe and exhale. After then, the patient was told to let go of the leg and relax the hips and leg so that it might fall to the floor. A light overpressure was placed to the foot while the patient relaxed and let their hip and leg fall farther to the ground. Five times a day, this exercise was performed with a two-minute break in between each set.
Instability of pelvic girdle	To make pelvic girdle stable	1.Straight leg raise: Patient position: Patient will be in supine lying Therapist position: By the affected side of patient and stabilizes anterior superior iliac spine of the same side Procedure: Ask the patient to lift the leg upwards Set: 10 reps x 1 set Therabands are used while giving SLR so that strengthening of rectus femoris, core muscles and iliacus happens that help in providing pelvic girdle stability.
Reduced hand grip strength	To improve hand griping	Transcutaneous electrical nerve stimulation (TENS) therapy for 15 mins has reported increased hand grip strength.
Reduced muscle strength	To improve muscle strength	For upper limb: Isometric exercises can be performed to target various muscles of the shoulder joint, elbow joint, wrist joint, hip joint, knee joint, and ankle joint. Theraband and dumbbels can be used for progression 10 reps x 1 set

Outcome measures

On Rheumatoid Arthritis Disease Activity Index (RADAI-5), pre and post-rehabilitation scores were 5.2 and 2.3 respectively. On the quality of life scale, pre and post-rehabilitation scores were 60 and 85 respectively. Table 4 and Table 5 demonstrate MMT for the bilateral lower limb (pre-rehabilitation and post-rehabilitation) and ROM for the bilateral lower limb (pre-rehabilitation and post-rehabilitation), respectively.

**Table 4:** Manual muscle testing (MMT) for the bilateral lower limb (pre-rehabilitation and post-rehabilitation)

	Pre-rehabilitation			Post-rehabilitation	
Joint	Right	left	Joint	Right	Left
Shoulder			shoulder		
Flexors	3 out of 5	3 out of 5	Flexors	4 out of 5	4 out of 5
Extensors	3 out of 5	3 out of 5	Extensors	5 out of 5	5 out of 5
Abductors	3 out of 5	3 out of 5	Abductors	5 out of 5	5 out of 5
Medial rotators	3 out of 5	3 out of 5	Medial rotators	5 out of 5	5 out of 5
Lateral rotators	3 out of 5	3 out of 5	Lateral rotators	5 out of 5	5 out of 5
Elbow			Elbow		
Flexors	3 out of 5	3 out of 5	Flexors	5 out of 5	5 out of 5
Extensors	3 out of 5	3 out of 5	Extensors	5 out of 5	5 out of 5
Wrist			Wrist		
Flexors	3 out of 5	3 out of 5	Flexors	5 out of 5	5 out of 5
Extensor	2 out of 5	2 out of 5	Extensors	4 out of 5	4 out of 5
Radial deviators	3 out of 5	3 out of 5	Radial deviators	5 out of 5	5 out of 5
Ulnar deviators	3 out of 5	3 out of 5	Ulnar deviators	5 out of 5	5 out of 5
Hip			Hip		
Flexors	3 out of 5	3 out of 5	Flexors	4 out of 5	4 out of 5
Extensors	3 out of 5	3 out of 5	Extensors	5 out of 5	5 out of 5
Abductors	3 out of 5	3 out of 5	Abductors	4 out of 5	4 out of 5
Adductors	3 out of 5	3 out of 5	Adductors	5 out of 5	5 out of 5
Medial rotators	3 out of 5	3 out of 5	Medial rotators	5 out of 5	5 out of 5
Lateral rotators	3 out of 5	3 out of 5	Lateral rotators	5 out of 5	5 out of 5
Knee					
Flexors	3 out of 5	3 out of 5	Flexors	5 out of 5	5 out of 5
Extensors	3 out of 5	3 out of 5	Extensor	5 out of 5	5 out of 5
Ankle					
Dorsiflexors	3 out of 5	3 out of 5	Dorsiflexors	5 out of 5	5 out of 5
Plantarflexors	3 out of 5	3 out of 5	plantarflexors	5 out of 5	5 out of 5
Invertors	2 out of 5	3 out of 5	Invertors	4 out of 5	4 out of 5
Evertors	2 out of 5	2 out of 5	Evertors	4 out of 5	4 out of 5

**Table 5:** Range of motion (ROM) for the bilateral lower limb (pre-rehabilitation and post-rehabilitation).

	Pre Rehabilitation			Post rehabilitation	
Joint	Right	left	Joint	Right	Left
Shoulder			shoulder		
Flexion	0^0^-120^0^	0^0^-90^0^	Flexion	0^0^-160^0^	0^0^-154^0^
Extention	0^0^-60^0^	0^0^-50^0^	Extention	0^0^-60^0^	0^0^-60^0^
Abduction	0^0^-124^0^	0^0^-110^0^	Abduction	0^0^-170^0^	0^0^-160^0^
Medial rotation	0^0^-70^0^	0^0^-55^0^	Medial rotation	0^0^-70^0^	0^0^-60^0^
Lateral rotation	0^0^-80^0^	0^0^-70^0^	Lateral rotation	0^0^-90^0^	0^0^-80^0^
Elbow			Elbow		
Flexion	0^0^-120^0^	0^0^-120^0^	Flexion	0^0^-145^0^	0^0^-150^0^
Extension	120-0^0^	120-0^0^	Extension	145^0^-0^0^	150^0^-0^0^
Wrist			Wrist		
Flexion	0^0^-70^0^	0^0^-65^0^	Flexion	0^0^-120^0^	0^0^-135^0^
Extention	90-0^0^	135-0^0^	Extension	120^0^-0^0^	135^0^-0^0^
Radial deviation	0^0^-15^0^	0^0^-15^0^	Radial deviation	0^0^-20^0^	0^0^-20^0^
Ulnar deviation	0^0^-20^0^	0^0^-25^0^	Ulnar deviation	0^0^-30^0^	0^0^-30^0^
Hip			Hip		
Flexion	0^0^-80^0^	0^0^-70^0^	Flexion	0^0^-120^0^	0^0^-120^0^
Extension	0^0^-24^0^	0^0^-20^0^	Extension	0^0^-30^0^	0^0^-30^0^
Abduction	0^0^-40^0^	0^0^-35^0^	Abduction	0^0^-45^0^	0^0^-40^0^
Adduction	0^0^-20^0^	0^0^-20^0^	Adduction	0^0^-30^0^	0^0^-30^0^
Medial rotation	0^0^-35^0^	0^0^-25^0^	Medial rotation	0^0^-45^0^	0^0^-40^0^
Lateral rotation	0^0^-35^0^	0^0^-20^0^	Lateral rotation	0^0^-45^0^	0^0^-40^0^
Knee					
Flexion	0^0^-90^0^	0^0^-80^0^	Flexion	0^0^-135^0^	0^0^-135^0^
Extension	90-0^0^	80-0^0^	Extension	135-0^0^	135-0^0^
Ankle					
Dorsiflexion	0^0^-10^0^	0^0^-15^0^	Dorsiflexion	0^0^-20^0^	0^0^-20^0^
Plantarflexion	0^0^-25^0^	0^0^-30^0^	plantarflexion	0^0^-50^0^	0^0^-50^0^
Inversion	0^0^-20^0^	0^0^-25^0^	Inversion	0^0^-35^0^	0^0^-35^0^
Eversion	0^0^-10^0^	0^0^-10^0^	Eversion	0^0^-15^0^	0^0^-15^0^

## Discussion

The significance of a multidisciplinary approach in the treatment of individuals with intricate autoimmune disorders is highlighted by this instance. In order to treat the systemic symptoms linked to SS (Sjogren’s syndrome) as well as the articular and periarticular symptoms associated with RA, physiotherapy was essential. The patient's functional status and quality of life improved overall as a result of the customized physiotherapy therapies that improved joint mobility, decreased discomfort, and strengthened muscle. The results of the study showed a statistically significant reduction in anterior pelvic tilting angle, a notable improvement in post-intervention values for sacroiliac mobility, and a statistically significant decrease in pain in Mulligan mobilization with movement group hence, For the treatment of patients with persistent sacroiliac joint problems, Mulligan mobilization combined with movement is a more successful approach [16]. It was discovered that Mulligan mobilization was more beneficial for alleviating sacroiliac joint discomfort [17]. For patients with RA, laser treatments significantly reduce discomfort and enhance function right away [18].

A key component of physiotherapy care for RA patients is disease management. Physiotherapists instruct patients in joint protection techniques, assistive device use, and therapeutic exercise performance in collaboration with occupational therapists [14]. Increasing exercise and/or physical activity can have a positive, simultaneous effect on several RA systemic signs and disease-related symptoms [19]. Extended duration of moderate-to-intense land-based aerobic and muscle-strengthening exercises lowers activity barriers and enhances muscular strength and oxygen absorption [20]. Significant improvement was observed in the post-rehabilitation period, as indicated by the outcome measures of RADAI-5 and quality of life. The patient's range of motion of joints and muscle strength also showed positive changes after the physiotherapy interventions. According to some scientists, rheumatoid arthritis exercises enhance general health, lessen pain, increase mobility, support the preservation of joint function, have a positive impact on the heart and general circulation, and ultimately enhance patients' quality of life. Therapeutic physical culture, which is frequently utilized in RA with a variety of unique clinical symptoms, continues to be one of the most significant approaches to physical rehabilitation [21]. In order to effectively manage patients with autoimmune rheumatic disorders, rheumatologists, physiotherapists, and other healthcare professionals must continue to collaborate, as this instance highlights. It draws attention to the possible advantages of physiotherapy for people with complicated autoimmune diseases like RA and SS in terms of symptom relief, improved physical function, and general well-being. Furthermore, the favorable results noted in this instance bolster the inclusion of physiotherapy as a crucial part of the all-encompassing care given to patients suffering from autoimmune rheumatic disorders.

## Conclusions

This case report presented the significance of adopting a multidisciplinary strategy in the treatment of intricate autoimmune conditions like RA and SS. The patient was diagnosed with both RA and SS and exhibited a range of symptoms affecting various joints and extra-articular organs, leading to a decline in her quality of life. The comprehensive physiotherapy interventions tailored to address the specific needs of the patient played a pivotal role in improving her functional status and overall well-being. The physiotherapeutic goals focused on educating the patient about the recuperation process, managing physiological and psychological agony, and employing various interventions to address specific issues. The patient's range of motion of joints and muscle strength also showed positive changes, highlighting the efficacy of physiotherapy in managing autoimmune rheumatic disorders. The positive outcomes observed in this case support the integration of physiotherapy as an integral component of the overall care plan for patients with autoimmune rheumatic disorders. 
